# Lensed Chemically Etched Optical Fibers for Free-Space Coupling of Multicore Fibers

**DOI:** 10.3390/ma19051013

**Published:** 2026-03-06

**Authors:** Arkadiusz Woźniak, Mateusz Łakomski, Przemysław Niedzielski

**Affiliations:** Department of Semiconductor and Optoelectronic Devices, Lodz University of Technology, Politechniki 8 Ave., 93-590 Lodz, Poland; mateusz.lakomski@p.lodz.pl (M.Ł.); przemyslaw.niedzielski@p.lodz.pl (P.N.)

**Keywords:** free-space coupling, optical fiber, multicore fiber

## Abstract

The present study introduces a novel design for lensed, chemically etched optical fibers (LEOFs) designed for efficient coupling with multicore fibers (MCFs). Experimental characterization at a wavelength of 1550 nm yielded an average coupling loss of approximately 0.76 dB under direct physical contact and 0.40 dB when the fiber end was positioned at an optimal working distance. Moreover, it was experimentally demonstrated that LEOFs exhibit high tolerance to longitudinal displacement and minimal wavelength-dependent variation in coupling efficiency. Based on this approach, fiber-in–fiber-out (FIFO) multicore couplers were fabricated using bundles of LEOFs that had been aligned to MCF cores. Bidirectional measurements yielded average insertion losses of 3.23–3.30 dB in TX and 3.20–3.27 dB in RX transmission directions at 1550 nm, with core-resolved losses as low as 1.09 dB for well-aligned channels. The results confirm the viability of LEOF-based multicore free-space coupling, with further improvements expected from enhanced fabrication accuracy.

## 1. Introduction

The increasing demands on optical communication systems, as a result of global data traffic, have prompted the exploration of space-division multiplexing (SDM) technologies as a promising approach to overcoming the fundamental capacity limitations of conventional single-mode fiber systems [1,2]. SDM allows for the simultaneous transmission of multiple spatial channels within a single optical fiber, thereby substantially increasing transmission capacity without the necessity for additional spectral resources [3]. In the realm of diverse SDM implementations, multicore fibers have emerged as one of the most promising platforms, as they integrate multiple independent cores within a single cladding, enabling parallel data transmission while preserving compatibility with existing fiber infrastructure [4]. Recent advancements in multicore fiber design and fabrication have enabled enhanced core density, reduced inter-core crosstalk, and augmented system integration capabilities [5]. Furthermore, multicore fibers have been demonstrated to enable record-breaking transmission capacities, thus confirming their potential as a key technology for next-generation high-capacity optical communication systems [6]. A pivotal step in the practical deployment of MCFs is the realization of efficient fan-in/fan-out (FIFO) devices. Backward compatibility with telecom single-mode fibers (SMFs) is also important. Depending on the network application, the bend-insensitive, large effective area or non-zero dispersion-shifted optical fibers are typically utilized [7]. The primary challenge pertains to the achievement of achieving low-loss, low-crosstalk coupling between individual SMFs and the closely spaced cores of an MCF. Two primary strategies have been pursued in the literature: contact- and non-contact (free-space)-based couplers. Contact-based coupling approaches generally depend on precise physical alignment and structural modification of fiber assemblies. For instance, V-groove substrate-based FIFO devices enable accurate positioning of individual SMFs relative to the MCF cores, providing stable and reproducible coupling performance [8]. Similarly, fiber bundle-type fan-out structures permit simultaneous interfacing of multiple SMFs with multicore fibers, thus offering a scalable solution for multicore connectivity [9,10]. More recently, advanced FIFO configurations and MCF couplers based on direct structural integration and optimized core matching have been demonstrated, achieving improved optical performance and reduced insertion loss [11,12]. Nevertheless, contact couplers have inherent limitations as fiber tapering alters the core size and may require customized refractive index profiles. Moreover, high-precision splicing or capillaries are necessary to maintain rotational alignment [13]. An alternative approach concentrates on the utilization of glass waveguides as coupling elements between MCF and SMFs [14]. This way, the optical path conversion from two-dimensional core arrangements into a one-dimensional array can be achieved. Another technology that is being intensively developed involves spot size converts (SSCs) [15]. Such structures including thermally expanded core fibers, adiabatic tapers, and integrated photonic edge couplers have demonstrated very low coupling losses even below 0.2 dB [16]. These solutions have been shown to be particularly effective in fiber-to-chip and waveguide coupling applications, where permanent structural modification of the waveguide or fiber is feasible. However, their application to MCF coupling presents significant challenges due to the need for precise and simultaneous mode matching across multiple closely spaced cores, as well as limited flexibility once fabricated.

On the other hand, non-contact couplers based on free-space optics have attracted growing attention because they overcome many of these limitations [17,18,19,20]. In such schemes, the optical field emerging from the SMF is collimated or focused by a microlens, including a GRIN lens [18], allowing efficient mode matching to the target MCF core without physical contact. This approach offers several advantages: it provides greater tolerance to variations in fiber geometry and allows additional optical elements—such as filters, isolators [17], or beam splitters—to be integrated in the coupling path [21,22]. Free-space coupling is also inherently flexible, making it suitable for interfacing with MCFs of different layouts or for hybrid configurations involving SMFs, MCFs, and multimode fibers. However, these benefits come at the cost of increased sensitivity to alignment accuracy and mechanical stability, as the lens position and focal distance must be tightly controlled.

In this context, chemically etched and lensed fibers represent a particularly promising implementation of non-contact coupling. Hydrofluoric acid (HFA) etching enables precise reduction in the cladding diameter while leaving the fiber core intact, thus minimizing propagation losses [23,24,25]. As demonstrated in our previous study, which employed utilizing chemical etching techniques, the low insertion loss and crosstalk suppression were found to be below 0.4 dB and −51.3 dB, respectively [13]. Subsequent formation of spherical microlenses at the etched optical fiber tips allows the emitted light to be focused on a tailored working distance, directly onto the MCF cores. This strategy combines the low-loss potential of etched fibers with the flexibility of free-space optics, offering a compact, scalable, and mechanically robust pathway towards the practical realization of MCF fan-in/fan-out devices.

In this work, we present a fabrication method for lensed etched optical fibers (LEOFs), which are optimized for free-space coupling into MCFs. Standard SMFs are etched from 125 µm down to approximately 40 µm in diameter, after which spherical microlenses are thermally formed at their tips. The investigation encompasses the geometrical precision and optical performance of the resulting structures, alongside the evaluation of insertion losses. In addition, the proposed LEOFs are assembled into fiber bundles to form a FIFO multicore coupler, thereby enabling a direct experimental verification of the coupling concept at the device level. The fabricated FIFO structures are characterized bidirectionally at 1550 nm in order to assess insertion loss, optical symmetry, and core-to-core uniformity. This established a direct link between single-fiber performance and practical multicore coupling.

## 2. Materials and Methods

### 2.1. LEOF Idea

**Figure 1:**
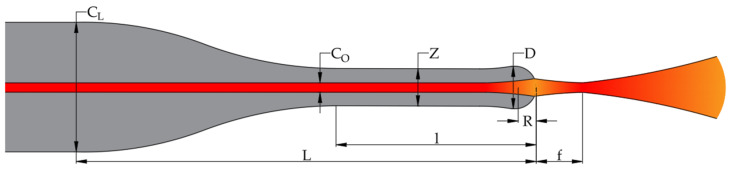
The idea of lensed tapered optical fiber.

The LEOF concept is presented in Figure 1, where the geometrical variables are typical for lensed type optical fibers [26]. The LEOF is based on an etched optical fiber (EOF) terminated with a lens structure. The diameter (*Z*) and length (*l*) of the EOF, as well as the lens diameter (*D*) and curvature radius (*R*) of the LEOF, can be arbitrarily adjusted depending on the application. Consequently, the parameter *L* denotes the distance measured from the tip of the LEOF to the axial position at which the fiber diameter reaches the standard value of 125 µm. The LEOF focuses light on a focal point (*f*) that is located at a focal length ranging from 15 µm to 40 µm from the forehead of the LEOF. A bundle of such optical fibers can be used for multicore fiber coupling, thereby providing a non-contact solution and enabling integration with other optical elements.

### 2.2. Fabrication

**Figure 2:**
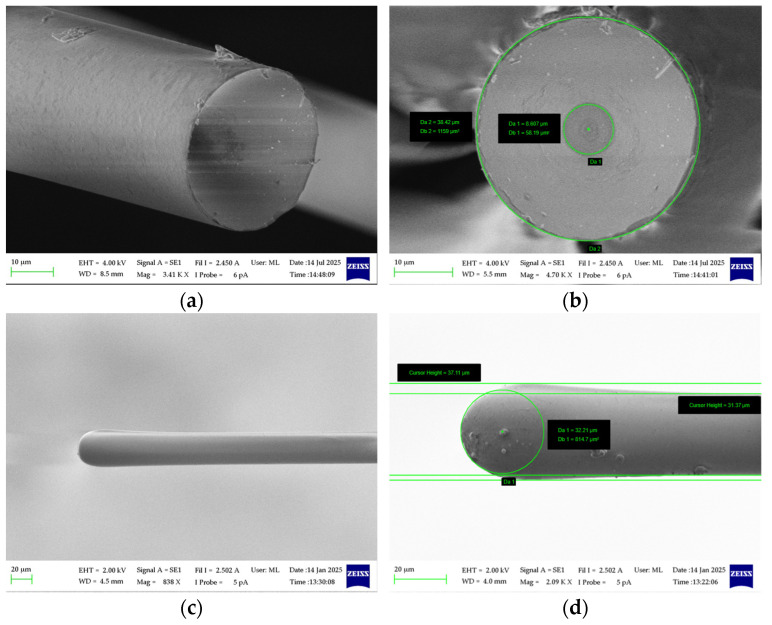
Silica-based glass optical fiber after HF etching process (**a**), forehead view (**b**), fabricated lensed optical fiber (**c**), and its geometrical characterization (**d**).

In order to reduce the diameter of optical fibers, the process of chemical etching was carried out in HFA solution. HFA is a strong acid with the capacity to etch into a variety of materials, with glass being particularly susceptible to its effects. Over the years it has been proven that it can also be used for etching of optical fibers [27,28,29]. In the present study, optical fibers were etched in a 50% HFA solution at a temperature of 20.5 °C for a duration of 22.5 min. The action of the acid was neutralized by 30 min of immersion in 1 M sodium hydroxide (NaOH) and subsequently 30 min in H_2_O, respectively [13]. Following the HFA etching process, the optical fiber is characterized by a concave tip profile as presented in Figure 2a,b. It is presumed that this concavity results from the lower bond energy of Ge–O and the presence of defects [30].

Chemical etching of the optical fiber selectively is known to result in a selective reduction in the diameter of the cladding while preserving the core size. Because the process mainly removes cladding material, the guidance of the fundamental mode is only slightly affected. Consequently, this etched region typically results in minimal additional insertion loss [1]. Moreover, the diameter change as a function of etching time demonstrates a high degree of linearity, thus facilitating effective process control.

Etched single-mode optical fibers were used for the fabrication of microlenses directly at their end faces. The lens formation process was achieved through the localized melting of the fiber tip surface, a process that was executed using a customized fusion program that was implemented on a Fujikura (Tokyo, Japan) S152 fusion splicer. The process was specifically designed to facilitate controlled material reflow, resulting in the formation of a smooth, axially symmetric microlens structure. The developed method eliminates the need for mechanical cleaving or precision cutting of the concave end face of etched optical fibers. It is particularly advantageous to avoid such mechanical post-processing when working with small-diameter or chemically etched fibers, where conventional cleaving introduces a significant risk of fracture, surface defects, or misalignment. Consequently, this approach provides a reliable and repeatable technique for fabricating high-quality microlenses on etched fibers while simplifying the overall manufacturing process and improving yield.

To achieve a microlens with an optimal and highly symmetric geometry, the ratio between the lens diameter and the etched optical fiber diameter should be maintained at a minimum value of approximately 1.13. When the diameter of the lens falls below this threshold, irregularities and air inclusions within the lens tend to arise. This phenomenon can be attributed to the residual concave morphology in the core region formed during the HFA etching process as presented in Figure 2a. Such imperfections have the potential to compromise the optical performance by introducing scattering losses and compromising the focusing characteristics of the lens. Excessively oversized lenses may result in additional optical attenuation, primarily due to increased sensitivity to lateral or angular misalignments during fiber-to-fiber coupling. The curvature of the lens surface was characterized using scanning electron microscopy (SEM) followed by circular fitting of the spherical tip profile. Moreover, the lens diameter of each LEOF was verified prior to use. For lenses with a nominal diameter of 37 µm, the measured dimensional deviation remained within the range of +0.36/−0.83 µm among more than 200 fabricated lenses. An example of fabricated LEOF based on the above assumptions is presented in Figure 2c. As can be seen in Figure 2d, two distinct parameters of the LEOF can be distinguished: the lens diameter *D* and the radius of curvature *R*. For the fabricated samples of LEOFs, these parameters were measured as follows: 20 µm (10 µm), 30 µm (15 µm), 37 µm (30 µm), 40 µm (32.5 µm), 65 µm (40 µm), 90 µm (47.5 µm).

## 3. Simulations

### 3.1. Model

In order to optimize the design of a lensed fiber for efficient free-space coupling, a series of numerical simulations was conducted. The primary objective of the analysis was to determine the optimal lens geometry that maximizes coupling efficiency and to evaluate the impact of longitudinal and lateral misalignment on coupling efficiency. The propagation of light from the lensed, chemically etched fiber towards the receiving fiber was modeled using a scalar diffraction framework implemented in the Python 3.10 programming language. The algorithm represents the optical field on a two-dimensional Cartesian grid and propagates it using the Angular Spectrum Method (ASM). This approach is based on the decomposition of the optical field into plane waves in the spatial frequency domain, providing an exact solution to the scalar Helmholtz equation, which is well suited for modeling light propagation in systems where diffraction is essential. Polarization effects, material dispersion, and birefringence were neglected, as the weakly guiding, near-Gaussian modes and the relatively low numerical aperture of the system ensure that the scalar approximation remains highly accurate.

The multicore fiber specifications were implemented in the simulation model with a core diameter of 8.2 µm, a refractive index of 1.45173 for the core, and 1.446 for the cladding, based on standard SMF fiber optic reflective index simulation findings [31], these parameters correspond to those of the produced samples. Furthermore, the refractive index of the lens was determined through a weighted average of 1.44746, based on the composition of the cladding and core material at the end of the etched fiber being processed. The LEOF was modeled with spherical geometries corresponding to the experimental fabrication results shown in Figure 2. The investigation encompasses lens diameters of 20 µm, 30 µm, 37 µm, 40 µm, 65 µm, and 90 µm, with corresponding radii of curvature of 10 µm, 15 µm, 32.5 µm, 40 µm, 47.5 µm, and 75 µm, thereby ensuring comprehensive coverage of the entire spectrum of fabricated samples. In order to guarantee a high level of numerical accuracy, the simulation domain was defined as a 120 × 120 µm^2^ windows, which was the discretized into a 2048 × 2048 point grid. This resolution effectively suppresses numerical dispersion and aliasing errors, allowing for precise evaluation of the beam evolution and the resulting coupling efficiency. Furthermore, given that the lens diameter is significantly larger than the operating wavelength (1550 nm). Consequently, the scalar treatment provides an adequately accurate description of the diffraction phenomena.

### 3.2. Light Propagation and Beam Characterization

The light source was modeled as a fundamental LP_01_ mode of a single-mode fiber core, approximated by a Gaussian beam with a waist radius (*w*_0_) of 5.2 µm. The amplitude distribution of the electric field can be described by

(1) $$E(r,0)=E_{0}exp(-\frac{r^{2}}{w_{0}^{2}})$$

where

$r=\sqrt{x^{2}+y^{2}}$—radial distance from the optical axis.

*E*_0_—peak amplitude.

The lens was modeled as a phase-shifting element located at the fiber tip. The phase delay *Φ*(*r*) was calculated based on the local thickness of the lens profile, effectively transforming the fundamental fiber mode into a converging or diverging wavefront. Assuming a spherical lens, the phase transformation is given by

(2) $$\Phi \left(r\right)=k_{0}(n_{lens}-n_{air})(\sqrt{R^{2}-r^{2}}-R)$$

where

$k_{0}=2\pi /\lambda$—the vacuum wavenumber.

*n_lens_* and *n_air_*—the refractive indices of the lens and the surrounding medium.

*R*—the radius of curvature of the lens.

The simulation model accounts for the physical aperture of the fiber tip, Fresnel reflection losses at the lens–air interface, and the diffractive propagation of the wavefront towards the receiving fiber. The key performance metrics investigated are the coupling efficiency (*η*) into a receiving fiber and insertion loss (*IL*). The coupling capabilities of the system are calculated using the Overlap Integral. This method is employed to calculate the overlap between the propagated field *E_prop_* at a given distance *z* and the fundamental mode of the receiving fiber *E_ref_*:

(3) $$\eta (z)=\frac{{|\iint E_{prop}(x,y,z)E_{ref}^{*}(x,y)dxdy|}^{2}}{\iint |E_{prop}(x,y,z){|}^{2}dxdy\cdot \iint |E_{ref}(x,y){|}^{2}dxdy}\cdot T_{Fresnel}$$

The total insertion loss can be calculated as

(4) $$IL\left[dB\right]=-10log10(\eta )$$

### 3.3. Results

**Figure 3:**
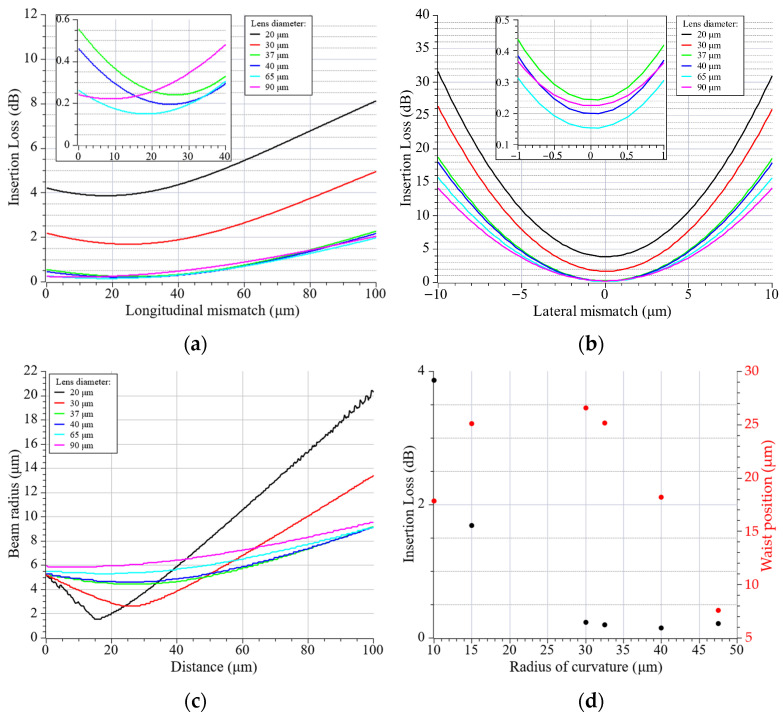
Numerical analysis results of the lensed fiber performance: (**a**) insertion loss as a function of longitudinal mismatch and (**b**) lateral mismatch for different lens diameters; beam radius change along the propagation distance (**c**) and impact of the lens radius of curvature on the minimum insertion loss (black) and beam waist position (red) (**d**).

**Figure 4:**
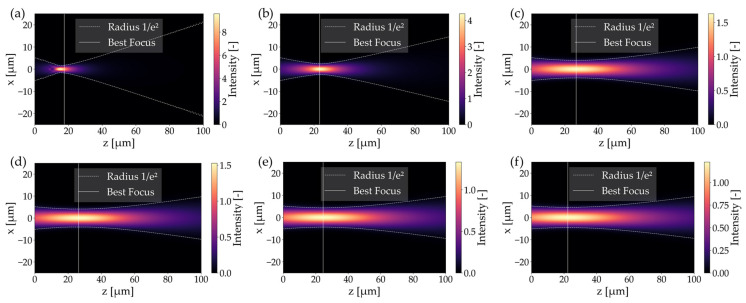
Cross-sectional illumination fields for (**a**) 20 µm, (**b**) 30 µm, (**c**) 37 µm, (**d**) 40 µm, (**e**) 65 µm, and (**f**) 90 µm lens diameter with indicated focal point (white line) and beam diameter (white dotted line).

As illustrated in Figure 3a, the simulation results of insertion loss as a function of longitudinal mismatch, comparing six lens diameters: 20 µm, 30 µm, 37 µm, 40 µm, 65 µm, and 90 µm with radius curvatures of 10 µm, 15 µm, 32.5 µm, 40 µm, 47.5 µm, and 75 µm. A clear dependence between the lens size and focal depth can be observed. Lensed fibers with a lens diameter greater than 65 µm have been shown to produce a significantly flatter response, thereby offering greater tolerance to axial positioning errors. It has been demonstrated that lenses with a diameter of 20 µm and 30 µm exhibit a reduced optimal working distance, indicating higher sensitivity to longitudinal misalignment. As shown in Figure 3c, small-diameter lenses produce a very narrow beam waist of 2 µm approximately at a focal length of 17.8 µm and 25.1 µm, respectively. While this results in high peak intensity (Figure 4a), the rapid divergence and significant Mode Field Diameter (MFD) mismatch with the receiving fiber leads to higher baseline losses. Most notably, 37 µm, 40 µm, and 65 µm lenses demonstrated optimal performance achieving the lowest minimum losses within the 0.15–0.24 dB range. Quantitatively, the theoretical longitudinal tolerance (±Δz) for a power degradation of <0.5 dB was extracted from the simulation data. For lenses with a diameter ranging from 37 to 65 μm, the tolerance falls within the range of ±33 μm to ±39.7 μm, exhibiting an increase in proportion to the lens diameter. The 90 μm lens exhibits a longitudinal tolerance of ±45 μm, whereas for the 20 μm lens, it is significantly narrower at ±22 μm, highlighting the increased stability for larger lens geometries. Figure 3b depicts the sensitivity to lateral misalignment (offset along the *x*-axis). The response exhibited a parabolic trend, with smaller diameter lenses exhibiting steeper curves, indicating stricter alignment tolerances. A misalignment of 5 µm in lateral orientation was observed, resulting in insertion losses ranging from 3.6 dB to 10.4 dB for lensed fibers considered in this study. The beam radius results presented in Figure 3c demonstrate that the beam radii for lenses in the 37–90 µm range are comparable, varying between 4.5 and 5.8 µm at the waist position. To further evaluate the system’s robustness, the theoretical lateral tolerance (±Δx) for a <1 dB loss penalty was determined. The 37 μm lens provides a lateral tolerance of ±2.53 μm, while the 20 μm lens requires a much stricter alignment of ±1.91 μm. Similarly to the longitudinal tolerance, in this analysis, increase in this parameter with larger lens diameter is visible. This two-fold increase in tolerance justifies the use of larger lenses to ease the assembly requirements of the FIFO device. Taking this observation into account, together with the IL and waist position results from Figure 3d, it can be confirmed that optimal coupling occurs when the lens curvature generates a mode field that closely matches the MFD parameter of the receiver. Therefore, 37–65 μm diameter range is a relevant consideration.

In order to facilitate a more profound comprehension of the correlation between insertion loss and waist size in a lensed fiber, the numerical analysis was extended to monitor the longitudinal intensity distribution (x-z plane). The results of this study are shown in Figure 4, which provides a visual representation of the beam’s propagation behavior as it exits the lens. The white solid line indicates the position of maximum intensity (best focus), whilst the dotted white lines represent the 1/e^2^ beam width. For the 20 µm and 30 µm diameter lenses (Figure 4a), strong focusing can be observed, resulting in a short focal length and a high numerical aperture of the beam. This accounts for the higher insertion losses due to the significant mode field mismatch. The utilization of larger lenses results in the beam starts to elongate, thereby leading to a reduction in divergence. The beam geometry becomes more favorable for efficient mode coupling. The 90 µm lens (Figure 4f) presents a semi-collimated beam with a weak focus, resulting in higher insertion loss compared to the optimum obtained for the lenses in the 37–65 µm diameter range.

**Figure 5:**
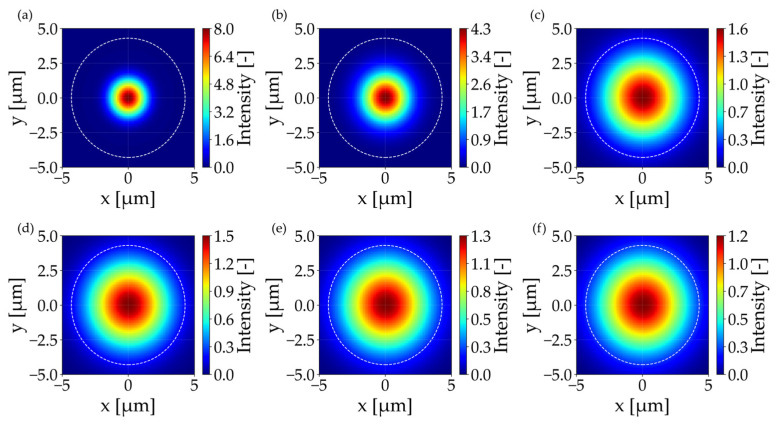
Perpendicular illumination fields for (**a**) 20 µm, (**b**) 30 µm, (**c**) 37 µm, (**d**) 40 µm, (**e**) 65 µm, and (**f**) 90 µm lens diameter with receiving fiber core size (white dotted line).

The effect of different lens diameters on coupling efficiency can be best understood by analyzing the intensity profiles at the point of minimum insertion loss. Figure 5 presents the perpendicular illumination fields (x-y plane) at the best focus for each considered lens. The white dotted circle on each graph represents the core of the receiving fiber, serving as a spatial reference for the Mode Field Diameter (MFD) of the fundamental mode. As analyzed before, 20 µm and 30 µm lenses generate highly concentrated spots with a peak intensity of approximately 8 a.u. However, the spot size is smaller than the receiving mode area. This mismatch, combined with the phase mismatch resulting from the high beam NA, leads to the high insertion losses observed in Figure 3a. Lensed fibers with lens diameter above 37 µm produce spot sizes that overlap with the receiving fiber mode area, but with much lower peak intensity (1.2–4.3 a.u.) due to energy being distributed over a larger effective area. The lens with a diameter of 90 µm produces a waist broader than the receiving core, resulting in a significant amount of energy falling outside the guided mode acceptance area, which increases the coupling loss.

## 4. Experimental Results

### 4.1. Measurement Setup

**Figure 6:**
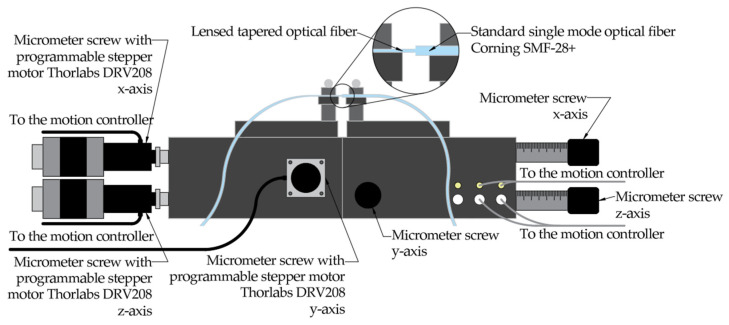
Measurement setup of the coupling loss between lensed tapered optical fiber and SMF 28e+ fiber.

The experimental measurement setup, schematically illustrated in Figure 6, was constructed to enable high-precision characterization of optical coupling between LEOFs and a standard single-mode optical fiber. The alignment platform consisted of a Thorlabs (Newton, MA, USA) MAX373K2/M three-axis flexure stage equipped with closed-loop piezoelectric actuators, providing sub-micrometer positioning resolution and excellent mechanical stability. In addition to the primary alignment stage, a secondary positioning system was employed, consisting of a Thorlabs (Newton, MA, USA) MAX303/M micrometric stage. The apparatus was equipped with high-precision micrometric screws with Thorlabs (Newton, MA, USA) DRV-208 integrated stepper motors, enabling automated, computer-controlled adjustment of the stage position along three orthogonal axes. The motion control system was based on Thorlabs (Newton, MA, USA) BSC203 three-channel stepper motor controlled by custom-developed software, ensuring high repeatability and automated operation with sub-micrometer positioning accuracy. The EXFO (Quebec, QC, Canada) FLS-600 was utilized as the laser source, with the optical power being measured using the integrated Thorlabs (Newton, MA, USA) NTA007 photodetector of the Thorlabs (Newton, MA, USA) BNT001 controller.

**Figure 7:**
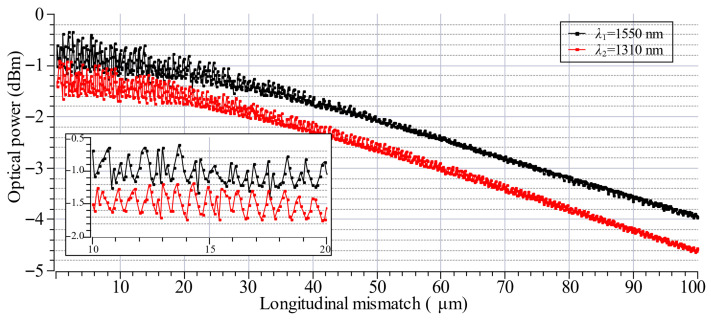
Signal interference in relation to the longitudinal mismatch between optical fibers.

During the experimental characterization, optical signal interference effects were observed, as illustrated in Figure 7. This phenomenon is most pronounced at small longitudinal offsets. As the distance between the optical fibers increases, the relative amplitude of these fluctuations decreases, leading to a gradual reduction in the power fluctuations between successive measurement points. To mitigate these fluctuations and the resulting measurement instability, a statistical averaging strategy based on 100 independent measurements per point was implemented. By averaging the transmission loss values obtained from multiple independent trials, the influence of random interference artifacts was minimized, ensuring that the reported insertion loss values more accurately reflect the intrinsic optical performance of the fiber system.

### 4.2. Lens Fabrication Repeatability

**Figure 8:**
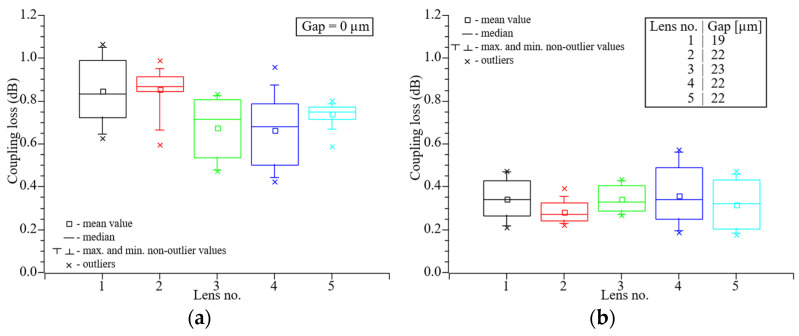
Coupling loss of the connection between lensed etched optical fiber and cleaved SMF-28e+ fiber. Lens diameter equals to 40 µm. Gap between fibers: 0 µm (**a**), optimal working distance (**b**).

To verify the repeatability of the lens fabrication process we used five randomly selected lensed etched optical fibers fabricated by using the same splicing program. In this experiment the lens diameter was equal to 40 µm with 32.5 µm curvature radius. The measurements were performed bidirectionally for direct contact between fibers and at an optimal working distance corresponding to the smallest coupling loss. As seen in Figure 8a, direct connection of the lensed fibers leads to relatively high insertion loss. This is a result of a low stiffness of a small-diameter fiber which leads to difficulty with creation of a strong direct connection and hence air gaps and misalignments between fibers. This phenomenon can be eliminated by a bundle of LEOFs, which increase stiffness and hence misalignment resistance. The average coupling loss was 0.76 dB, whereas differences in average attenuation between individual lenses is on relatively high, ranging from 0.42 to 1.07 dB. Figure 8b presents the optimal working distance, with the average coupling loss decreasing and reaching 0.39 dB. Moreover, for such distance between fibers, reduction in the interference can be observed. This leads to much lower differences between the highest and the lowest signal levels, which is in the range from 0.18 to 0.65 dB.

**Figure 9:**
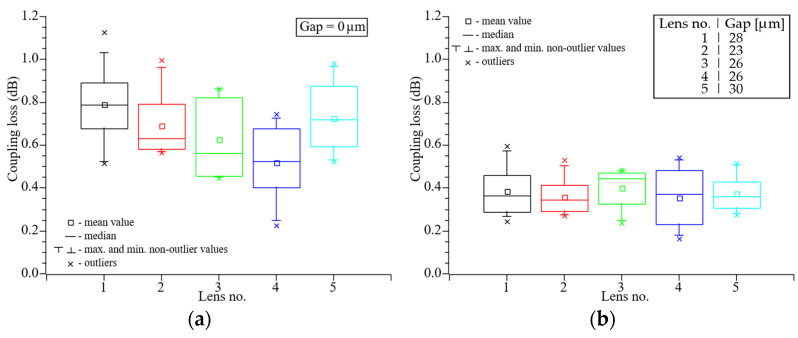
Coupling loss of the connection between cleaved SMF-28e+ fiber and lensed etched optical fiber. Lens diameter equals to 40 µm. Gap between fibers: 0 µm (**a**), optimal working distance (**b**).

A comparable coupling efficiency was achieved for the configuration with the LEOFs on the receiver side (Figure 9). However, greater variability was observed between individual lenses. Nevertheless, the results obtained are promising and suggest potential for further improvements. The average coupling loss was around 0.67 dB, while the attenuation values exhibited significant dispersion, ranging from 0.23 to 1.13 dB. At the optimal working distance shown in Figure 9b, the average coupling loss decreased to 0.40 dB. Moreover, the difference between the highest and lowest signal levels was reduced, with the measured values falling within the range of 0.17–0.60 dB.

### 4.3. Longitudinal Mismatch

**Figure 10:**
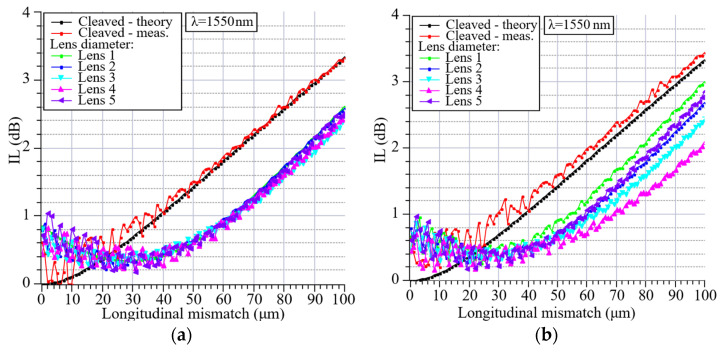
Influence of a distance between the 40 µm diameter LEOF and cleaved SMF-28e+ fiber on the coupling loss. LEOF transmission direction TX (**a**), RX (**b**).

We also examined the influence of longitudinal misalignment on the average coupling loss between the LEOFs with a 40 µm lens diameter and cleaved SMF-28e+ fiber. The distance between the fibers was varied in the range of 0–100 µm. We measured five randomly selected LEOFs fabricated with the same splicing program. All the measurements were performed bidirectionally at a wavelength of 1550 nm. Moreover, we compared the obtained results with a configuration consisting of two cleaved SMF-28e+ fibers and with their theoretically predicted coupling loss. As can be seen in Figure 10, for the cleaved single-mode fibers, the attenuation increases consistently with the distance between the fiber end faces. Such a correlation is expected due to the reduction in coupled optical power with growing separation. In the case of LEOFs, a different behavior was observed. For small separations, the attenuation initially decreased, reaching a minimum in the range of 20–30 µm depending on the lens and transmission direction. This separation corresponds closely to the optimal working distance of the lensed fiber, where the optical mode is best matched to the core of the receiving fiber. For larger separations, a gradual increase in attenuation was observed, resulting from an increased mode field diameter beyond the optimal working distance. These results confirm that for lensed fibers, optimal coupling occurs near the focal point, while for cleaved fibers, efficient coupling is maintained only under direct physical contact. The use of LEOFs can enhance coupling efficiency for larger distances between fibers and reduce the impact of longitudinal misalignments, which is beneficial in free-space multicore coupling systems. A comparison of the transmission direction does not indicate any major differences, but more pronounced influence of the signal interference at small fibers separations and greater variations between lenses for longer distances are observed for the RX transmission direction.

In order to verify the occurrence of chromatic aberration in LEOFs, measurements were taken for 1550 nm and 1310 nm sources. These were then compared to the simulation results, the details of which can be found in Table 1. The difference between simulated and experimental insertion loss was found to be less than 0.07 dB at 1310 nm and 0.03 dB at 1550 nm, which corresponds to an approximate coupling efficiency difference of less than 2%. The discrepancy between the simulated and experimental working distances can be attributed to the influence of positioning inaccuracies and geometrical tolerances. However, both distances remain consistent with each other when considering the effect of the wavelength employed. By comparing the minimum coupling loss and the corresponding working distance for both wavelengths, Table 1 provides a quantitative assessment of the axial chromatic aberration of the system as 3.8 µm. Moreover, numerical calculation demonstrates that, for the specified system parameters, i.e., size, fiber core size, mode field diameter, lens diameter and curvature, spherical aberration remains a marginal factor. It is evident that the radial extent of the incident Gaussian beam (w_0_ = 4.6 µm) occupies a negligible fraction of the total lens aperture (D = 37 µm). Consequently, the beam propagates predominantly through the paraxial zone (Figure 4). In this localized central region, the deviation of the spherical surface from the ideal parabolic profile is negligible (0.33%). Consequently, the phase distribution is not subject to significant higher-order distortions, ensuring that the focal spot characteristics and the fundamental mode coupling efficiency are governed primarily by diffraction limits rather than geometric aberrations.

### 4.4. Multicore Optical Fiber Coupling

**Figure 11:**
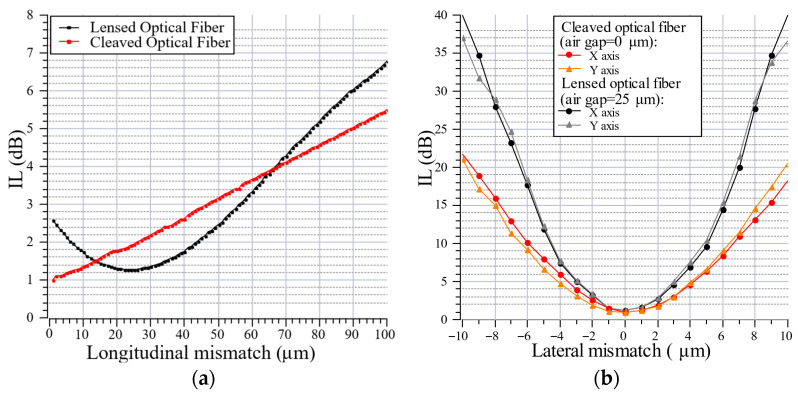
The characteristics of longitudinal (**a**) and lateral (**b**) mismatch losses of connection between lensed or cleaved type of optical fiber and multicore optical fiber.

In the next step, the lensed optical fiber with a lens diameter of 37 µm and a curvature radius of 30 µm was used to measure the longitudinal and lateral mismatch losses when coupled into a SiliSeven MCF produced by Silitec (Boudry, Switzerland). The diameter of the lens was selected to correspond to the spatial arrangement of the multicore fiber, which features a core-to-core pitch of 37 µm. As illustrated in Figure 11a, the insertion loss (IL) is presented as a function of longitudinal mismatch between two optical fibers. For the cleaved optical fiber, the insertion loss increases almost linearly with increasing air gap. The initial value of 0.99 dB is higher than in the previously described case, due to the geometrical core mismatch between SMF and MCF. However, the lensed optical fiber still exhibits a minimum insertion loss equal to 1.27 dB at a finite longitudinal separation, corresponding to the effective working distance of the lensed fiber concept. At a distance of 25 µm, the focused beam waist is optimally matched to the diameter of the receiving central core of the MCF, thereby leading to enhanced coupling efficiency. These results clearly demonstrate that the lensed fiber enables non-contact coupling, provided that the axial separation is maintained within a relatively narrow optimal range. Additionally, Figure 11b shows the insertion loss as a function of lateral mismatch for both cleaved and lensed fibers. All measured characteristics exhibit a symmetric, parabolic dependence with a minimum at zero offset. However, a significant difference in sensitivity to lateral displacement is apparent. The lensed optical fiber shows a much steeper increase in insertion loss with increasing lateral mismatch compared to the cleaved fiber, indicating a substantially reduced alignment tolerance. This increased sensitivity arises from the focusing properties of the lensed optical fiber, which reduces the beam waist at the coupling plane. While a smaller spot size minimizes insertion losses, it also leads to a rapid degradation of the coupling efficiency when lateral misalignment is introduced. In contrast, the cleaved fiber produces a larger beam spot, which is predictable due to its numerical aperture, and results in greater tolerance to lateral offsets.

**Figure 12:**
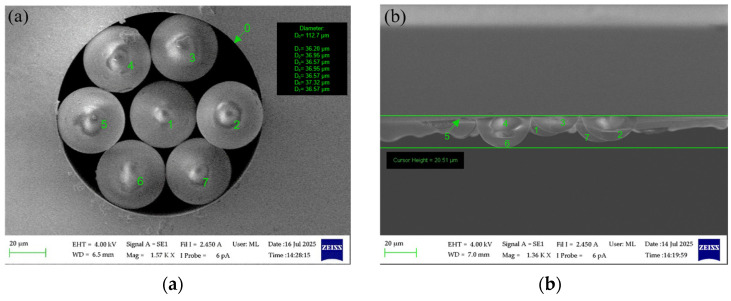
Fabricated multicore FIFO device based on the bundle of lensed, etched optical fibers: forehead (**a**), top view (**b**).

To fabricate a multicore FIFO device based on the 37 µm diameter LEOFs, we used a custom ceramic ferrule produced by Adamant Namiki Precision Jewel Co., Ltd. (Tokyo, Japan), now operating as Orbray Co., Ltd. (Tokyo, Japan) [32]. The inner diameter of the ferrule, equal to 111 µm, corresponds to three times the core-to-core distance of the SiliSeven multicore optical fiber. The FIFO device was fabricated by placing seven LEOFs inside the ferrule. As shown in Figure 12a,b, the fabricated FIFOs are characterized by a non-uniform fiber spacing. Several factors contribute to this effect. First, the LEOFs exhibit non-uniform diameters, ranging from 36.2 µm to 37.32 µm. Although these differences are relatively small, they lead to the formation of gaps between adjacent microlenses. As a result, lateral fiber displacement occurs, leading to an irregular fiber arrangement within the bundle. The variations in microlens diameter result from the limited suitability of the optical fiber fusion splicer used for thermal processing of fibers with such small diameters. Professional glass processing systems are expected to significantly reduce this variation. The second contributing factor to the irregular spacing between the LEOFs is the inner diameter of the ferrule exceeding the nominal value specified by the manufacturer. According to the datasheet, the nominal hole diameter should be within the range of 111.0–112.0 µm. However, SEM measurements revealed that the inner diameter of the ferrule used in this study was 112.7 µm. This enlarged inner diameter leads to increased free spaces between the fibers, which further aggravates the non-uniform of fiber positioning within the bundle and consequently limits the common overlap area of the cores between LEOFs and MCF.

The insertion loss measurements were performed by systematically aligning the outer cores of the FIFOs in all possible mutual configurations. This alignment procedure enabled identification of optimal coupling conditions corresponding to the minimum transmission loss for each pair of aligned cores. From the six available core alignment configurations, those exhibiting the lowest average insertion loss were selected for the final realization of all FIFOs, thereby reducing the impact of geometrical misalignment on the coupling efficiency.

The measurements were carried out in both the transmission (TX) and reception (RX) directions, utilizing a wavelength of 1550 nm. The results obtained are presented in Table 2 and show that the average insertion losses in the TX direction were 3.23 dB, 3.30 dB, and 3.23 dB for FIFO 1, FIFO 2, and FIFO 3, respectively. Correspondingly, the mean losses measured in the RX direction were 3.27 dB, 3.20 dB, and 3.20 dB, respectively. The close agreement between the TX and RX averages indicates good optical symmetry of the fabricated structures and confirms the absence of significant direction-dependent coupling effects. A more detailed analysis of the individual cores reveals noticeable variations in insertion loss between channels. The lowest measured losses, reaching 1.09 dB (TX, FIFO 1, core 2) and 1.19 dB (RX, FIFO 3, core 2), indicate highly efficient coupling and good geometrical alignment for selected core configurations. In contrast, the highest recorded losses exceed 6 dB, particularly for core 4 in FIFO 2 and FIFO 3, which points to substantial core-to-core misalignment and reduced mode overlap. The relatively high standard deviation values, ranging from approximately 1.15 dB to 1.69 dB, confirm a significant non-uniformity of coupling performance across individual cores within the same FIFO. This effect is especially pronounced for FIFO 2 and FIFO 3, where the largest insertion loss fluctuations are observed. These findings indicate that, despite selecting the optimal alignment configurations, the spatial configuration of the LEOFs does not align precisely with the cores of the MCF.

The higher-than-expected insertion loss can be mainly attributed to fiber displacements, irregular arrangement of the LEOFs within the bundle, and imperfect matching between LEOF positions and the cores of the multicore fiber. In future work, improvements to the fabrication process are scheduled, with a particular emphasis on more precise lens diameter and hence enhanced control over the bundle geometry. These improvements are expected to reduce the insertion loss spread among individual cores and lower the maximum loss values, bringing the overall FIFO performance closer to or below that of the best-performing channels.

To measurement of inter-core crosstalk, we used a method based on Fresnel reflection at the glass–air interface [33]. Through this approach, light at a wavelength of 1550 nm is launched into a single channel of the FIFO, while the optical power reflected from the adjacent cores is analyzed to determine the inter-channel crosstalk level. The method offers high measurement sensitivity and enables crosstalk evaluation without the need for an additional FIFO device to extract signals from individual cores of the multicore fiber. By comparing the Fresnel-reflected power from the excited core with that from adjacent cores, the crosstalk can be directly derived from the measured power difference, with common Fresnel reflection losses canceling out. Using this methodology, the inter-core crosstalk of the fabricated FIFOs was measured. The measurements were performed for selected cores under identical excitation conditions to ensure consistency and comparability of the results. The obtained crosstalk values, calculated from the measured reflected optical powers, are summarized and presented in Table 3.

The obtained results indicate that the inter-core crosstalk levels of all the FIFOs do not exceed −46.25 dB, while the average crosstalk value is −57.23 ± 3.89 dB. Such low crosstalk levels clearly confirm the high optical channel isolation. Furthermore, a comparative analysis of different FIFO samples revealed no statistically significant differences in the measured crosstalk values, demonstrating the high and repeatability and robustness of the core isolation achieved in FIFO devices based on bundles of LEOFs.

## 5. Conclusions

We demonstrated the coupling efficiency of lensed etched optical fibers, fabricated by chemical etching combined with electrical arc processing using a fusion splicer. Numerical simulations revealed that the coupling performance is critically dependent on the lens geometry. A lens diameter in the range of 37 µm to 65 µm was identified as an optimal design, resulting in theoretical insertion losses ranging from 0.16 to 0.24 dB. In addition, functional optimal working distance for coupling lances fibers with selected lens diameters was estimated to be between 20 and 26.6 µm from the lens tip.

Experimental results confirmed that the fabricated lensed, chemically etched optical fibers are characterized by low insertion loss and high resistance to longitudinal displacement. Measurements performed at a wavelength of 1550 nm showed that the average coupling loss was approximately 0.76 dB when the fiber end faces were in direct physical contact, and 0.40 dB near the optimal working distance of the lenses. The obtained results suggest that lensed, chemically etched fibers are a promising solution for free-space coupling applications, particularly in MCF-based systems and compact photonic interconnects.

A FIFO device based on bundles of LEOFs was fabricated and experimentally evaluated. Bidirectional measurements, performed in both transmission and reception directions at 1550 nm, yielded average insertion losses of 3.23–3.30 dB (TX) and 3.20–3.27 dB (RX), confirming good optical symmetry and the absence of direction-dependent coupling effects. Individual core analysis revealed insertion losses as low as 1.09 dB for well-aligned channels, demonstrating that highly efficient multicore coupling is achievable using the proposed approach. At the same time, significant loss variations between individual cores were observed, with maximum losses exceeding 6 dB and standard deviations ranging from approximately 1.15 dB to 1.69 dB. This non-uniformity is primarily attributed to residual fiber displacements, irregular spatial arrangement of the LEOFs within the bundle, and imperfect matching between the LEOF positions and the multicore fiber geometry.

In addition to insertion loss performance, inter-core crosstalk was experimentally investigated to assess channel isolation in the fabricated FIFO devices. The measured crosstalk levels did not exceed −46.25 dB, with an average value of −57.23 ± 3.89 dB, confirming excellent optical isolation between adjacent cores. Such low crosstalk levels indicate that the proposed LEOF-based FIFO effectively suppresses unwanted inter-channel coupling, despite the observed non-uniformities in insertion loss. These results confirm that, from the perspective of inter-core isolation, the developed multicore coupling approach is well suited for high-density MCF-based photonic interconnects.

The proposed LEOF-based coupling approach offers the advantages of simple fabrication, compact form factor, and the ability to achieve low insertion loss without the need for additional free-space optics. However, it also exhibits several important limitations. In multicore configurations, the performance is further limited by channel-to-channel non-uniformity arising from residual misalignments, fabrication tolerances, irregular bundle geometry, and imperfect matching between the LEOF positions and the multicore fiber layout. These factors cause the overall FIFO performance to be dominated by the worst-performing channels. Additionally, the coupling characteristics represent an effective near-field optimum rather than a diffraction-limited focus, limiting direct interpretation using classical lens models. The approach therefore involves a trade-off between efficiency, alignment tolerance, scalability, and fabrication complexity, while long-term stability, environmental robustness, and reproducibility in high-density bundles remain key challenges for practical use.

Overall, the presented results demonstrate the feasibility of LEOF-based coupling for multicore fiber systems and highlight both the potential and current limitations of the approach. Future work will focus on improving fabrication precision through enhanced control of fiber positioning and bundle geometry, optimizing lens parameters for multicore alignment, and systematically evaluating long-term stability and environmental robustness. Moreover, the utilization of surrounding mediums that differ from air, such as index-matching gel or photocured-resin [34], may also improve coupling efficiency. These improvements are expected to reduce channel-to-channel loss variation, lower maximum insertion losses, and bring the overall FIFO performance closer to or exceed that of the best-performing individual channels, enabling practical deployment in high-density photonic interconnects.

## Figures and Tables

**Table 1:** Simulated and experimental coupling parameters of LEOF coupling at 1310 nm and 1550 nm, illustrating chromatic aberration and validating the numerical model.

	Parameter	1550 nm	1310 nm
Simulation	Min. coupling loss [dB]	0.24	0.38
	Working distance [µm]	26.32	24.56
	Beam Waist w_0_ [µm]	4.43	3.59
	Waist Position z_0_ [µm]	26.60	24.62
Experiment	Min. coupling loss [dB]	0.31	0.41
	Working distance [µm]	24.20	20.60

**Table 2:** Insertion loss of fabricated FIFOs based on lensed chemically etched optical fibers.

Insertion Loss *λ* = 1550 nm (dB)						
	TX			RX		
Core No	FIFO 1	FIFO 2	FIFO 3	FIFO 1	FIFO 2	FIFO 3
1	3.13	2.16	2.94	2.98	1.95	2.81
2	1.09	2.27	1.24	1.56	1.86	1.19
3	4.02	5.03	3.44	4.05	4.96	3.20
4	2.07	6.08	6.18	2.22	6.16	6.41
5	4.35	2.30	3.06	4.23	2.33	3.30
6	3.12	2.96	1.92	3.07	3.04	1.80
7	4.84	2.29	3.80	4.77	2.13	3.70
Avg.	3.23	3.30	3.23	3.27	3.20	3.20
Max.	4.84	6.08	6.18	4.77	6.16	6.41
Min.	1.09	2.16	1.24	1.56	1.86	1.19

**Table 3:** Inter-core crosstalk of fabricated FIFOs based on lensed chemically etched optical fibers.

Inter-Core Crosstalk *λ* = 1550 nm (dB)			
	FIFO 1	FIFO 2	FIFO 3
Avg.	−57.55	−56.70	−57.45
Max.	−49.13	−46.25	−48.39
Min.	−65.19	−65.56	−62.80

## Data Availability

The original contributions presented in this study are included in the article. Further inquiries can be directed to the corresponding author.

## Abbreviations

The following abbreviations are used in this manuscript:

LEOF	Lensed chemically etched optical fiber
MCF	Multicore fiber
TX	Transmission
RX	Reception
SDM	Space-division multiplexing
FIFO	Fan-in/Fan-out device
SMF	Single-mode optical fiber
HFA	Hydrofluoric acid
SEM	Scanning electron microscopy
FWHM	Full width at half maximum
IL	Insertion loss
ASM	Angular Spectrum Method
MFD	Mode Field Diameter
